# Longitudinal Study of Boxing Therapy in Parkinson’s Disease, Including Adverse Impacts of the COVID-19 Lockdown

**DOI:** 10.21203/rs.3.rs-355283/v1

**Published:** 2021-04-07

**Authors:** Craig Horbinski, Katelyn B. Zumpf, Kathleen McCortney, Dean Eoannou

**Affiliations:** Northwestern University; Northwestern University; Northwestern University; Parkinson’s Boxing

**Keywords:** Boxing therapy, Parkinson Disease, COVID-19

## Abstract

**Background:**

Parkinson’s Disease (PD) is a highly prevalent neurodegenerative disease whose incidence is increasing with an aging population. One of the most serious manifestations of PD is gait instability, leading to falls and subsequent complications that can be debilitating, even fatal. Boxing therapy (BT) uses gait and balance exercises to improve ambulation in people with PD, though its efficacy has not yet been fully proven.

**Methods:**

In the current longitudinal observational study, 98 participants with idiopathic PD underwent twice-weekly BT sessions. Primary outcome was self-reported falls per month; secondary outcomes were quantitative and semi-quantitative gait and balance performance evaluations. Statistical methods included segmented generalized estimating equation with an independent correlation structure, binomial distribution, and log link.

**Results:**

The average number of self-reported falls per month per participant decreased by 87%, from 0.86 ± 3.58 prior to BT, to 0.11 ± 0.26 during BT. During the lockdown imposed by COVID-19, this increased to 0.26 ± 0.48 falls per month. Females and those > 65 years old reported the greatest increase in falls during the lockdown period. Post-lockdown resumption of BT resulted in another decline in falls, to 0.14 ± 0.33. Quantitative performance metrics, including standing from a seated position and standing on one leg, largely mirrored the pattern of falls pre- and post-lockdown.

**Conclusions:**

BT may be an effective option for many PD patients.

## Background

Parkinson’s Disease (PD) is among the most common neurodegenerative conditions worldwide, with an estimated lifetime risk of 2% and a prevalence of 1–2 per 1000 people over 65 years old [1]. Although not directly lethal, the motor disturbances and gait imbalance caused by PD greatly increase the risk of falls, which in turn increases risk of head trauma, fractures, and subsequent immobility. That, in turn, increases risk of thrombosis and infections; indeed, the most common cause of death in PD patients is pneumonia [2]. Thus, therapeutic approaches designed to reduce the risk of falls can have a great impact on patients with PD.

Boxing therapy (BT)–specifically, key exercises employed by boxers to improve their balance and gait in the ring—is becoming a popular way to mitigate PD-associated motor deficits. Much of what has been published is qualitative in nature, describing overall satisfaction with BT programs by PD participants [3, 4]. However, empiric data supporting BT in PD, especially its ability to reduce the risk of falls, is scarce [5]. Studies that do contain specific, rigorous, quantitative data on BT were based on cohorts of ~ 30 participants or less [6, 7].

In this study, we explored the effects of BT in a cohort of 98 participants with PD, focusing on risk of falls as a primary endpoint. Because longitudinal tracking of BT participants encompassed the COVID-19 pandemic-associated lockdown, the effect of that lockdown, and the associated temporary cessation of BT, on outcomes is also addressed.

## Methods

### BT exercises and performance measurements

On initial evaluation, each participant was screened for details regarding their PD diagnosis, including symptoms, self-reported frequency of falls, other medical conditions and comorbidities, and medications. Each participant was then matched with a trainer, who provided one-on-one assessment and coaching throughout the duration of the program. Twice per week, each participant worked with their trainer on specific boxing-related exercises aimed at improving overall coordination, gait, and balance.

At the beginning of each month, including before the very first session, each participant was asked to estimate how many falls they had experienced the prior month. At that time, each participant was evaluated by their trainer as follows: (i) the number of times a participant could stand up from a sitting position on a chair in 15 seconds; (ii) the number of seconds, up to 30, that a participant could stand on one leg before losing balance.

Participants were also evaluated as to how well they could stand up from the floor, their normal walk, their ability to walk a straight line, their ability to walk backwards, their ability to walk with one foot crossing over in front of the other foot, and heel-toe touching. For each of those parameters, a semi-quantitative scoring system was as follows:

1. participant was unable to perform the activity at all, even with help
2. participant required assistance 3 or more times, or stepped off the line 3 or more times in the case of heel-toe, crossovers, or walking a straight line
3. participant required no assistance

### Statistics

Descriptive statistics were used to summarize participant characteristics. Frequency and percent were recorded for all categorical variables and mean, standard deviation, median, inter-quartile range, and range for all numeric variables. To evaluate the change in risk of falling over the course of BT, the stay-at-home lockdown due to the COVID-19 pandemic, and return-to-BT periods, we performed a segmented Generalized Estimating Equation (GEE) with an independent correlation structure, binomial distribution, and log link. Models using different correlation structures (unstructured, exchangeable, auto-correlation, and m-dependent) and distributions (log-binomial or negative-binomial) were compared using QIC. We included a random effect for clients to account for repeat measurements. Fixed effects were specified according to Wagner et al. [8] with two change points. These include: (1) the month from baseline or BT onset; (2) a binary indicator for BT session due to the COVID-19 pandemic (coded 1 while BT was paused due to the pandemic and 0 otherwise); (3) the month since BT cessation (coded 0 if during the boxing or return period); (4) a binary indicator for return to BT (coded 1 after client returns to boxing after COVID-19 onset and 0 otherwise); (5) month since client returned to BT (coded 0 if during BT or BT cessation period). Additionally, we controlled for the average number of falls per month clients reported prior to BT. To determine whether the effect of BT was different between males and females, we repeated the analysis above within male and female sub-groups. Subgroup analyses were also performed on those < 65, 65–75, and >75 years of age.

To evaluate whether other performance measures, such as the number of times participants could stand upright from a chair or seconds participants were able to stand on their right and left leg, changed over time with the addition of BT, we again used segmented GEE models. A Poisson distribution with an autocorrelated correlation structure was used to model the number of times participants were able to stand from a chair. Analysis of the time participants were able to stand on their right and left leg was done in 2-fold: first, modelling the risk of standing for 30 seconds using a binomial distribution and log link, then modelling the number of seconds stood in those who could only stand less than 30 seconds using a Poisson distribution. In both cases, an independent correlation structure was assumed. Fixed effects included months from BT onset, and indicator for returning to BT after the COVID-19 lockdown, and the months since the client returned to BT.

No participants had performance measurements collected before BT began, nor during the COVID-19 lockdown. Few participants had performance measurements collected while BT was paused due to COVID-19, which would have otherwise served as a control state for participants. Thus, analysis was limited to data collected during BT before the lockdown and when participants returned to BT after the lockdown.

Analyses were performed using R (R Core Team, Version 2.0.3, 2020) and SAS software (Copyright (c) 2016 by SAS Institute Inc., Cary, NC, USA) and assumed a two-sided, 5% level of significance.

## Results

### Descriptive Statistics

Ninety-eight participants with idiopathic PD were enrolled in the study, with an average age of 70.6 years (Table 1). Twenty-two percent (22/98) of participants were female. Based on interviews conducted at the beginning of BT, participants self-reported an average of 0.86 ± 3.58 falls per month at baseline.

### Modeling Falls

**Figure 1:**
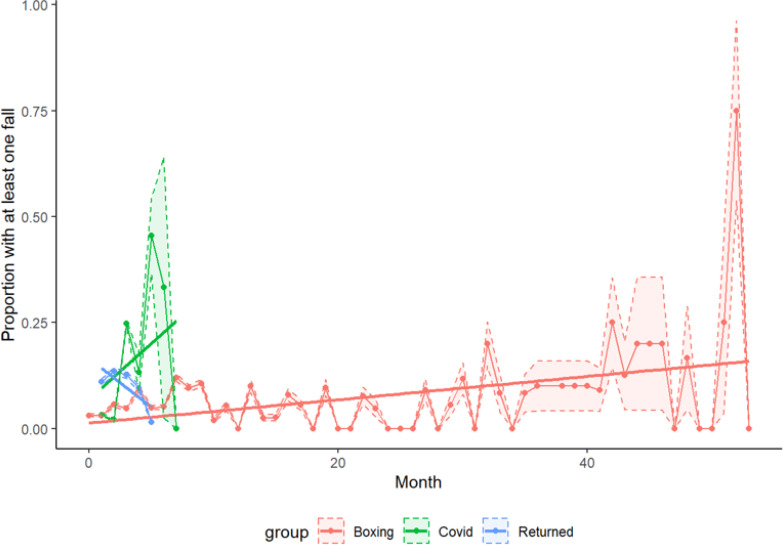
Proportion of participants who report at least one fall. Slope for each interval indicates change in relative risk over time.

Over 2,094 aggregate months of data, 175 falls were self-reported (8% of all participant-months). After adjusting for average self-reported number of falls per month prior to BT, there did not appear to be a significant change in relative risk of falling over time during the initial BT interval (RR: 1.01,95% CI (0.99, 1.03), *P*= 0.3050) (Table 3, Fig. 1, and Supplemental Table 1). From the beginning of the lockdown to the resumption of BT, the relative risk of falling increased by 51% each month (RRL 1.5058, 95% CI (1.26, 1.79), *P*< 0001). Once BT was resumed after the lockdown, the risk of falling decreased 20% each month (RR: 0.7992, 95% CI (0.68, 0.95), *P*= 0.0093); this was a 21% (RR: 0.79, 95% CI (0.67, 0.94), *P*= 0.0071) decrease in change in relative risk per month (i.e., slope) from the initial BT period and a 47% (RR: 0.53, 95% CI (0.41, 0.68), *P*< 0.001) decrease from the COVID-19 lockdown interval.

Similar results as described above were seen when excluding 49 participants who never reported any falls, at any time during the study period (Supplemental Tables 2 and 3).

### Sex as a Variable in Falls Reduced by BT

During the monitoring period, 10% (44/404) of female visits and 7% (131/1690) of male visits recorded a fall. In both males and females, there was no change in falls during the initial BT period or after returning post-lockdown. Similarly, females reported a greater increase in risk of falling per month during the COVID period: 84% increase per month in females (RR: 1.84, 95% CI (1.05, 3.24), *P*= 0.0318) vs. 49% increase per month in males (RR: 1.49, 95% CI (1.23, 1.81), *P*< 0.0001) (Table 4). After resuming BT, females experienced a 24% decrease in risk of falling per month (RR: 0.76, 95% CI (0.57, 0.996), *P*= 0.0471), whereas the decrease in males was not statistically significant (RR: 0.82, 95% CI (0.66, 1.02), *P*= 0.0718).

### Age as a Variable in Falls Reduced by BT

To assess the effect of age in BT and PD-associated falls, we stratified the cohort into participants < 65 years old (n = 20, 20%), 65–75 years old (n = 48, 49%), and >75 years old (n = 30, 31%) at the beginning of BT. Among those < 65 years old, 5% of all observations (22/445) reported a fall. This increased to 8% (88/1135) in those 65–75 years of age, and 9% (65/689) in those >75 years of age. Over the initial course of BT, risk of falls increased by 2% each month for those < 65 years old (RR: 1.02, 95% CI: (1.01,1.04), *P*= 0.0048) and >75 years old (RR: 1.02, 95% CI: (1.00, 1.05), *P*= 0.0405), but no significant change was observed in participants between 65–75 years (RR: 1.02, 95% CI: (0.98, 1.05), *P*= 0.2861) (Table 5). During the COVID-19 lockdown, those >65 years old experienced the greatest increase in the number of falls over time: 85% greater per month in those 65–75 years of age (RR: 1.85, 95% CI: (1.32, 2.59), *P*= 0.0003) and 59% greater in those greater than 75 years of age (RR: 1.63, 95% CI: (1.24, 2.15), *P*= 0.0005). There was no significant change in risk of falling in those< 65 years of age during the lockdown (RR: 1.74, 95% CI: (0.92, 3.28), *P*= 0.0903). As a result, only those 65–75 of age saw a significant reduction in risk of falling per month once BT was re-initiated after lockdown (RR: 0.76, 95% CI: (0.61,0.93), *P*= 0.0085).

### Performance Metrics

Other performance measures, such as the number of times participants were able to stand from a chair in 15 seconds, stand on each leg for 30 seconds, stand from the floor, walk normally, heel-toe touch, crossover, walk backwards, and, were collected for an average 16 ± 12 months of BT before the lockdown and 5 ± 1 months after resumption of BT. All metrics except standing from a chair and on each leg were semiquantitative, as described in the Methods. The median number of times participants were able to stand from a chair in 15 seconds was 7 times during BT and 8 times after the lockdown when BT resumed (Table 6). During the initial BT interval, participants were able to stand on their right and left leg for 15.7 ±11.3 and 14.7 ± 10.7 seconds, respectively. During the post-lockdown interval, the average duration increased to 17.1 ±11.4 and 16.2 ± 10.8 seconds on the right and left leg, respectively.

**Figure 2:**
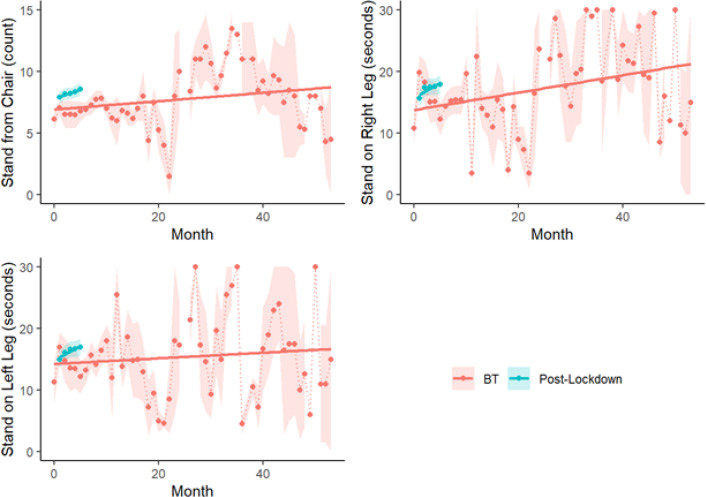
Distribution of performance measures over time by study interval. Slope for each interval indicates change in relative risk over time.

During the initial pre-lockdown BT period, the number of times participants were able to stand upright from a sitting position in a 15-second interval significantly increased over time (IRR: 1.01,95% CI: (1.00, 1.01), *P*= 0.0193) (Fig. 2). Likewise, after participants returned to BT post-lockdown, there was another significant improvement each month (IRR: 1.02, 95% CI: (1.01,1.04), *P*= 0.0014).

Regarding standing on one leg, of the 1,955 tests performed, 12% (n = 236) resulted in participants able to stand on their right leg for the full 30 seconds and 10% (n = 198) resulted in participants able to stand on their left leg for the full 30 seconds. There was no change in the odds of being able to stand on either leg over time, whether before the COVID-19 lockdown (Right leg RR: 1.01,95% CI (0.99, 1.03), *P*= 0.3544; Left leg OR: 1.01,95% CI: (0.98, 1.03), *P*= 0.5763) or after (Right leg RR: 1.02, 95% CI (0.96, 1.08), *P*= 0.5354; Left leg OR: 1.03, 95% CI: (0.96, 1.10), *P*= 0.4301). Of those that stood for less than 30 seconds, there was not a significant change in number of seconds standing over time on either leg before the lockdown (Right leg IRR: 1.00, 95% CI (0.99, 1.01), *P*= 0.9733; Left leg IRR: 1.00, 95% CI: (0.99, 1.01), *P*= 0.8169) (Fig. 2). However, after returning to BT after the lockdown, the average number of seconds standing on the right leg increased by 7% each month (IRR: 1.07, 95% CI: (1.00, 1.13), *P*= 0.0370). Similarly, the number of seconds standing on the left leg increased by 5% each month following the lockdown (IRR: 1.05, 95% CI: 1.00, 1.10), *P*= 0.0378).

Across both periods, the median semi-quantitative score that participants were able to stand from the floor, walk normally, heel toe touch, walk straight, or walk backwards was approximately 3/3 (Table 6). Because there was insufficient variance in those semi-quantitatively-scored metrics, no further analysis of those metrics was done.

## Discussion

Given the aging populations of the developed world, neurodegenerative conditions like PD are becoming more and more common. PD in particular is a problem of chronic risk management, especially reducing the risk of falls, since those falls often result in secondary trauma that is costly to manage, reduces quality-of-life, and increases mortality. Multiple studies have suggested that a variety of physical therapies and exercises can not only slow the rate at which the risk of falls increases in PD patients over time, but may also allow PD patients to regain some of what had been lost prior to initiation of therapy. For example, one meta-analysis suggested that many different approaches, including dancing, hydrotherapy, and robotic gait training, were very effective in PD patients, whereas the evidence for other therapies, like aerobics, Nordic walking, and BT, was less conclusive [9]. This is due, in large part, to the relative scarcity of studies that focus on a specific, rather unconventional therapeutic modality like BT [6, 7]. However, our data, in the largest cohort of its kind to date, suggest that BT may have value in improving gait stability and reducing the risk of falls in PD patients.

The COVID-19 pandemic has had a profound, deleterious, and long-lasting effect on populations worldwide. Even beyond those directly impaired or killed by the virus, measures that were implemented in an effort to slow the spread of disease, including the lockdown imposed by New York State from March-April 2020, have had severe economic and psychological repercussions. One of them is the delay or outright cancellation of health maintenance measures, such as well-visits and cancer screenings, that are critical for early prevention and/or detection of diseases that otherwise can quickly become unmanageable. The current data suggest this also holds true for interventions meant to reduce the impact of PD. In the current study, prior to the lockdown, the rate of self-reported falls had decreased by 87%, from 0.86 per month prior to initiation of BT down to 0.11 during BT (Table 1 and Table 2). But in that two-month lockdown, the rate of falls rapidly increased to more than double what had been observed during BT. Falls then declined again once BT was re-established, and eventually reached nearly the same level as before the lockdown. However, the rate at which improvements were regained after the lockdown was slower than the rate at which they were lost during the lockdown (Table 3). This is not surprising, and comports with other research on inactivity and muscle loss, including the deconditioning that happened during the COVID-19 pandemic [10]. In our cohort, the main risk factors for more rapid and severe increase in falls during BT cessation were female sex and participant age >65 years old (Table 4 and Table 5). In a prior study of exercise in PD, younger age and male sex were associated with better response to exercise [11]. Our current data comport with this, but also suggest that females can also reduce their risk of falls faster once BT is resumed.

Strengths of this observational study are its prospective nature and the relatively large cohort. One weakness is that the primary outcome, falls, relied on self-reporting, without any monitoring devices designed to record them. However, the quantitative performance metrics observed by a trainer, including standing from a chair and standing on one leg, largely matched the general patterns of what was reported for falls (Fig. 2). Another weakness is that this was not a randomized trial that compared BT to other PD therapies, or to a separate non-exercise control group. However, the COVID-19 lockdown inadvertently provided a unique opportunity to explore the time-dependent effect of BT cessation and resumption in the same cohort of participants. Furthermore, the statistical methods employed tracked each individual patient’s change over time, thereby correcting for other potential confounding variables.

## Conclusions

In sum, these data suggest that BT may reduce the risk of falls in PD patients. While other therapies also certainly have value, their ultimate utility depends greatly on the motivation and interest of the participant. BT might have a unique appeal because of its perceived novelty and association with a sport that has a very long history across numerous cultures.

## Supplementary Material

Supplement

## Figures and Tables

**Table 1:** Description of study participants.

	all (n = 98)
**age**	
mean (SD)	70.6 (7.98)
median (q1-q3)	71.0 (66.0–77.0)
[min, max]	[41.0, 89.0]
**age category**	
<65	20 (20.4%)
65–75	48 (49.0%)
>75	30 (30.6%)
**sex**	
male	76 (77.6%)
female	22 (22.4%)
**pre-BT self-reported average number of falls per month**	
mean (SD)	0.861 (3.58)
median (q1-q3)	0.0417 (0–0.167)
[min, max]	[0, 30.0]

The average follow-up interval during BT was 16.0 months (Table 2). During BT, the mean average number of self-reported falls per month per participant decreased from 0.86 ± 3.58 at baseline to 0.11 ± 0.26 during BT. During the COVID-19 lockdown, when BT was paused for an average of 3 months, falls increased from 0.11 ± 0.26 falls per month during the initial BT interval to 0.26 ± 0.48 falls per month. Once BT was resumed post-lockdown, participants reported another decline in falls, from 0.26 ± 0.48 falls per month to 0.14 ± 0.33. Likewise, the average proportion of months in which at least one fall was reported increased from 8 ± 0.15% during the initial phase of BT, to 12 ± 0.18% during the COVID-19 period, then decreased slightly to 10 ± 0.21 % after participants returned to boxing. (Of note, 17 of the original 98 participants did not resume BT after the lockdown.)

**Table 2:** Summary of falls per study interval.

	pre-lockdown BT (n = 98)	covid-19 lockdown interval (no BT) (n = 98)	post-lockdown BT (n = 81)
**months of follow-up**			
mean (SD)	16.0 (12.2)	3.22 (0.697)	4.73 (1.00)
median (q1-q3)	11.0 (8.25–21.8)	3.00 (3.00–3.00)	5.00 (5.00–5.00)
[min, max]	[2.00, 50.0]	[3.00, 7.00]	[1.00, 10.0]
**average number of falls per month**			
mean (SD)	0.109 (0.261)	0.255 (0.481)	0.143 (0.330)
median (q1-q3)	0 (0–0.106)	0 (0–0.333)	0 (0–0.200)
[min, max]	[0, 1.60]	[0, 2.00]	[0, 1.50]
**proportion of months with at least 1 fall**			
mean (SD)	0.0764 (0.151)	0.117 (0.182)	0.102 (0.207)
median (q1-q3)	0 (0–0.0890)	0 (0–0.333)	0 (0–0.200)
[min, max]	[0, 0.875]	[0, 0.800]	[0, 1.00]

Seventeen of the original 98 participants did not resume BT after the lockdown.

**Table 3:** Modelling risk of falling at least once, contrast estimates.

time period	change in risk over time			
	estimate	95% confidence interval		P
**BT**	1.0115	0.9897	1.0337	0.3050
**lockdown**	1.5058	1.2614	1.7975	<0.0001
**return**	0.7992	0.6750	0.9462	0.0093
**lockdown vs BT**	1.4887	1.2434	1.7825	<0.0001
**return vs lockdown**	0.5307	0.4148	0.6790	<0.0001

Modeling was based on the number of months in which at least one fall occurred. Return = post-lockdown resumption of BT.

**Table 4:** Sex as a variable in falls during BT, based on number of months in which at least one fall occurred.

	change in risk over time							
	females (n = 22)				males (n = 76)			
interval	estimate	95% confidence interval		P	estimate	95% confidence interval		P
**BT**	1.0224	0.9842	1.0621	0.2533	1.0103	0.9846	1.0366	0.4375
**lockdown**	1.8493	1.0549	3.2418	0.0318	1.4930	1.2311	1.8105	<0.0001
**return**	0.7559	0.5735	0.9964	0.0471	0.8202	0.6611	1.0177	0.0718
**lockdown vs BT**	1.8087	1.0532	3.1060	0.0317	1.4778	1.2147	1.7978	<0.0001
**return vs lockdown**	0.4088	0.2325	0.7186	0.0019	0.5494	0.4082	0.7394	<0.0001

Return = post-lockdown resumption of BT.

**Table 5:** Age as a variable in falls during BT, based on number of months in which at least one fall occurred.

interval	change in risk over time											
	< 65 years				65–75 years				>75 years			
	estimate	95% confidence interval		P	estimate	95% confidence interval		P	estimate	95% confidence interval		P
**BT**	1.0205	1.0062	1.0351	0.0048	1.0186	0.9847	1.0536	0.2861	1.0231	1.0010	1.0457	0.0405
**lockdown**	1.7353	0.9170	3.2837	0.0903	1.8494	1.3205	2.5903	0.0003	1.6311	1.2402	2.1452	0.0005
**return**	0.6626	0.4380	1.0021	0.0512	0.7553	0.6128	0.9309	0.0085	0.9374	0.6796	1.2929	0.6934
**lockdown vs BT**	1.7004	0.8988	3.2166	0.1027	1.8157	1.2865	2.5626	0.0007	1.5942	1.2111	2.0986	0.0009
**return vs lockdown**	0.3818	0.1901	0.7670	0.0068	0.4084	0.2713	0.6146	<0.0001	0.5747	0.3747	0.8813	0.0111

Return = post-lockdown resumption of BT.

**Table 6:** Summary of performance metrics.

	BT (n = 98)	post-lockdown (n = 81)	all (n = 179)
**months of follow-up**			
mean (SD)	16.0 (12.2)	4.73 (1.00)	10.9 (10.7)
median (q1-q3)	11.0 (8.25–21.8)	5.00 (5.00–5.00)	5.00 (5.00–11.5)
[min, max]	[2.00, 50.0]	[1.00, 10.0]	[1.00, 50.0]
**average number of falls per month**			
mean (SD)	0.109 (0.261)	0.143 (0.330)	0.124 (0.294)
median (q1-q3)	0 (0–0.106)	0 (0–0.200)	0 (0–0.118)
[min, max]	[0, 1.60]	[0, 1.50]	[0, 1.60]
**average stand from chair**			
mean (SD)	7.11 (3.47)	8.08 (3.97)	7.56 (3.73)
median (q1-q3)	7.25 (5.20–9.29)	7.80 (6.23–10.9)	7.55 (6.00–9.80)
[min, max]	[0, 14.0]	[0, 17.8]	[0, 17.8]
missing	11 (11.2%)	6 (7.4%)	17 (9.5%)
**average stand from floor**			
mean (SD)	2.61 (0.580)	2.68 (0.589)	2.64 (0.584)
median (q1-q3)	3.00 (2.00–3.00)	3.00 (2.78–3.00)	3.00 (2.00–3.00)
[min, max]	[1.00, 3.00]	[1.00, 3.00]	[1.00, 3.00]
missing	11 (11.2%)	6 (7.4%)	17 (9.5%)
**average normal walk**			
mean (SD)	2.99 (0.107)	2.98 (0.135)	2.98 (0.121)
median (q1-q3)	3.00 (3.00–3.00)	3.00 (3.00–3.00)	3.00 (3.00–3.00)
[min, max]	[2.00, 3.00]	[2.00, 3.00]	[2.00, 3.00]
missing	11 (11.2%)	6 (7.4%)	17 (9.5%)
**average heel toe touch**			
mean (SD)	2.47 (0.565)	2.47 (0.574)	2.47 (0.568)
median (q1-q3)	2.60 (2.00–3.00)	2.75 (2.00–3.00)	2.63 (2.00–3.00)
[min, max]	[1.00, 3.00]	[1.00, 3.00]	[1.00, 3.00]
missing	11 (11.2%)	6 (7.4%)	17 (9.5%)
**average crossovers**			
mean (SD)	2.41 (0.640)	2.49 (0.680)	2.45 (0.658)
median (q1-q3)	2.67 (2.00–3.00)	3.00 (2.00–3.00)	2.80 (2.00–3.00)
[min, max]	[1.00, 3.00]	[1.00, 3.00]	[1.00, 3.00]
missing	11 (11.2%)	6 (7.4%)	17 (9.5%)
**average walk straight line**			
mean (SD)	2.69 (0.510)	2.70 (0.486)	2.69 (0.498)
median (q1-q3)	3.00 (2.40–3.00)	3.00 (2.60–3.00)	3.00 (2.50–3.00)
[min, max]	[1.00, 3.00]	[1.00, 3.00]	[1.00, 3.00]
missing	11 (11.2%)	6 (7.4%)	17 (9.5%)
**average walk backwards**			
mean (SD)	2.82 (0.370)	2.77 (0.461)	2.80 (0.414)
median (q1-q3)	3.00 (2.90–3.00)	3.00 (2.80–3.00)	3.00 (2.80–3.00)
[min, max]	[1.00, 3.00]	[1.00, 3.00]	[1.00, 3.00]
missing	11 (11.2%)	6 (7.4%)	17 (9.5%)
**average stand on right leg**			
mean (SD)	15.7 (11.3)	17.1 (11.4)	16.3 (11.3)
median (q1-q3)	14.5 (4.00–27.1)	15.8 (7.20–30.0)	15.4 (5.21–29.4)
[min, max]	[0, 30.0]	[0, 30.0]	[0, 30.0]
missing	11 (11.2%)	6 (7.4%)	17 (9.5%)
**average stand on left leg**			
mean (SD)	14.7 (10.7)	16.2 (10.8)	15.4 (10.8)
median (q1-q3)	15.0 (4.30–26.3)	16.2 (6.10–27.3)	15.3 (4.85–26.6)
[min, max]	[0, 30.0]	[0, 30.0]	[0, 30.0]
missing	11 (11.2%)	6 (7.4%)	17 (9.5%)

SD = standard deviation.
