# Supplementary material for: Longitudinal Study of Boxing Therapy in Parkinson’s Disease, Including Adverse Impacts of the COVID-19 Lockdown
Source: Res Sq. 2021 Apr 7:rs.3.rs-355283. Preprint. [Version 1] doi: 10.21203/rs.3.rs-355283/v1 (PMC8043465; doi:10.21203/rs.3.rs-355283/v1)
Supplement: Supplement [file fc01e330cf6b3118e04a7f98.docx]

**Supplementary Data for**

**Longitudinal study of boxing therapy in Parkinson’s Disease, including adverse impacts of the COVID-19 lockdown**

Craig Horbinski,^1,2^ Katelyn B. Zumpf,^3^ Kathleen McCortney,^2^ and Dean Eoannou^4^

^1^Department of Pathology, Feinberg School of Medicine, Northwestern University, Chicago, IL 60611

^2^Department of Neurosurgery, Feinberg School of Medicine, Northwestern University, Chicago, IL 60611

^3^Department of Preventative Medicine, Feinberg School of Medicine, Northwestern University, Chicago, IL 60611

^4^Parkinson’s Boxing, Kenmore, NY 14217

| **parameter** |  | **estimate** | **standard error** | **95% confidence limits** | | **Z** | **Pr > \|Z\|** |
| --- | --- | --- | --- | --- | --- | --- | --- |
| **intercept** |  | -1.5905 | 0.4895 | -2.5499 | -0.6311 | -3.25 | 0.0012 |
| **self-reported average number of falls pre-BT** |  | 0.0378 | 0.0208 | -0.0030 | 0.0786 | 1.82 | 0.0693 |
| **months from baseline** |  | 0.0161 | 0.0119 | -0.0072 | 0.0394 | 1.35 | 0.1761 |
| **indicator for lockdown period** | 0 | 0.2072 | 0.3040 | -0.3886 | 0.8031 | 0.68 | 0.4955 |
| **indicator for lockdown period** | 1 | 0.0000 | 0.0000 | 0.0000 | 0.0000 | . | . |
| **months from start of lockdown follow-up** |  | 0.4180 | 0.0895 | 0.2426 | 0.5934 | 4.67 | <.0001 |
| **indicator for return to BT after lockdown** | 0 | -1.0523 | 0.3053 | -1.6507 | -0.4540 | -3.45 | 0.0006 |
| **indicator for return to BT after lockdown** | 1 | 0.0000 | 0.0000 | 0.0000 | 0.0000 | . | . |
| **months since return to BT after lockdown** |  | -0.2023 | 0.1048 | -0.4077 | 0.0032 | -1.93 | 0.0537 |

**Supplementary Table 1: Analysis of GEE Parameter Estimates: Empirical Standard Error Estimates.** Modeling was based on the absolute number of falls per month, excluding those who reported no falls at any time during the study period.

| **label** | **mean estimate** | **mean** | | **chi-square** | **Pr > ChiSq** |
| --- | --- | --- | --- | --- | --- |
|  |  | **confidence limits** | |  |  |
| **slope for BT** | 1.0162 | 0.9928 | 1.0402 | 1.83 | 0.1761 |
| **slope for lockdown** | 1.5436 | 1.2966 | 1.8375 | 23.82 | <.0001 |
| **slope for return** | 0.8301 | 0.6785 | 1.0157 | 3.27 | 0.0705 |
| **slope for return vs lockdown** | 0.5378 | 0.4088 | 0.7076 | 19.63 | <.0001 |
| **slope for lockdown vs BT** | 1.5189 | 1.2746 | 1.8102 | 21.82 | <.0001 |
| **slope for return vs BT** | 0.8169 | 0.6652 | 1.0032 | 3.72 | 0.0537 |

**Supplementary Table 2: Analysis of GEE Parameter Estimates: Contrast Estimate Results.** Modeling was based on the absolute number of falls per month, excluding those who reported no falls at any time during the study period.

| **parameter** |  | **estimate** | **standard error** | **95% confidence limits** | | **Z** | **Pr > \|Z\|** |
| --- | --- | --- | --- | --- | --- | --- | --- |
| **intercept** |  | -1.8763 | 0.4672 | -2.7920 | -0.9606 | -4.02 | <.0001 |
| **self-reported average number of falls pre-BT** |  | 0.0342 | 0.0173 | 0.0003 | 0.0681 | 1.98 | 0.0480 |
| **months from baseline** |  | 0.0092 | 0.0098 | -0.0099 | 0.0283 | 0.94 | 0.3474 |
| **indicator for lockdown period** | 0 | 0.4002 | 0.3390 | -0.2642 | 1.0647 | 1.18 | 0.2377 |
| **indicator for lockdown period** | 1 | 0.0000 | 0.0000 | 0.0000 | 0.0000 | . | . |
| **months from start of lockdown follow-up** |  | 0.3737 | 0.0926 | 0.1922 | 0.5553 | 4.03 | <.0001 |
| **indicator for return to BT after lockdown** | 0 | -1.1973 | 0.2931 | -1.7718 | -0.6227 | -4.08 | <.0001 |
| **indicator for return to BT after lockdown** | 1 | 0.0000 | 0.0000 | 0.0000 | 0.0000 | . | . |
| **months since return to BT after lockdown** |  | -0.2275 | 0.0874 | -0.3988 | -0.0562 | -2.60 | 0.0092 |

**Supplementary Table 3: Analysis of GEE Parameter Estimates: Empirical Standard Error Estimates.** Modeling was based on the number of months in which at least one fall occurred, excluding those who reported no falls at any time during the study period.

| **label** | **mean estimate** | **mean** | | **chi-square** | **Pr > ChiSq** |
| --- | --- | --- | --- | --- | --- |
|  |  | **confidence limits** | |  |  |
| **slope for BT** | 1.0092 | 0.9901 | 1.0287 | 0.88 | 0.3474 |
| **slope for lockdown** | 1.4665 | 1.2257 | 1.7547 | 17.50 | <.0001 |
| **slope for return** | 0.8038 | 0.6791 | 0.9516 | 6.44 | 0.0112 |
| **slope for return vs lockdown** | 0.5481 | 0.4260 | 0.7052 | 21.87 | <.0001 |
| **slope for lockdown vs BT** | 1.4531 | 1.2119 | 1.7424 | 16.28 | <.0001 |
| **slope for return vs BT** | 0.7965 | 0.6711 | 0.9453 | 6.78 | 0.0092 |

**Supplementary Table 4: Analysis of GEE Parameter Estimates: Contrast Estimate Results.** Modeling was based on the number of months in which at least one fall occurred, excluding those who reported no falls at any time during the study period.
